# Emergency Department Use by Released Prisoners with HIV: An Observational Longitudinal Study

**DOI:** 10.1371/journal.pone.0042416

**Published:** 2012-08-03

**Authors:** Jaimie P. Meyer, Jingjun Qiu, Nadine E. Chen, Gregory L. Larkin, Frederick L. Altice

**Affiliations:** 1 Department of Medicine, AIDS Program, Yale University School of Medicine, New Haven, Connecticut, United States of America; 2 Department of Emergency Medicine, Yale University School of Medicine, New Haven, Connecticut, United States of America; 3 Department of Surgery, Division of Emergency Medicine, University of Auckland School of Medicine, Auckland, New Zealand; 4 Department of Medicine, Division of Global Public Health, University of California San Diego School of Medicine, San Diego, California, United States of America; 5 Division of Epidemiology of Microbial Diseases, Yale University School of Public Health, New Haven, Connecticut, United States of America; University of Ottawa, Canada

## Abstract

**Background:**

Many people living with HIV access healthcare systems through the emergency department (ED), and increased ED use may be indicative of disenfranchisement with primary HIV care, under-managed comorbid disease, or coincide with use of other healthcare resources. The goal of this study was to investigate ED use by HIV-infected prisoners transitioning to communities.

**Methods:**

We evaluated ED use by 151 HIV-infected released prisoners who were enrolled in a randomized controlled trial of directly administered versus self-administered antiretroviral therapy in Connecticut. Primary outcomes were quantity and type of ED visits and correlates of ED use were evaluated with multivariate models by Poisson regression.

**Results:**

In the 12 months post-release, there were 227 unique ED contacts made by 85/151 (56%) subjects. ED visits were primarily for acute febrile syndromes (32.6%) or pain (20.3%), followed by substance use issues (19.4%), trauma (18%), mental illness (11%), and social access issues (4.4%). Compared to those not utilizing the ED, users were more likely to be white, older, and unmarried, with less trust in their physician and poorer perceived physical health but greater social support. In multivariate models, ED use was correlated with moderate to severe depression (IRR = 1.80), being temporarily housed (IRR = 0.54), and alcohol addiction severity (IRR = 0.21) but not any surrogates of HIV severity.

**Conclusions:**

EDs are frequent sources of care after prison-release with visits often reflective of social and psychiatric instability. Future interventions should attempt to fill resource gaps, engage released prisoners in continuous HIV care, and address these substantial needs.

## Introduction

People living with HIV/AIDS (PLWHA) present to the emergency department (ED) significantly more often than their HIV-uninfected counterparts. [1] This increased ED use, however, does not necessarily reflect severity of illness: ED visits among PLWHA, when compared to those without HIV, are no more likely to result in hospitalization. [1] Despite Department of Health and Human Services guidelines recommendation for continuous outpatient HIV follow-up, [2] many PLWHA frequently interface with healthcare systems through the ED.

Frequent and repetitive ED use represents excess cost to individuals and society in that most issues can often be addressed in primary care settings at lower premiums. A pattern of healthcare use with increased reliance on the ED may be emblematic of discontinuous primary care rather than more severe medical disease. [3], [4] On the other hand, some PLWHA who are engaged in HIV care may use the ED based on the recommendation of their HIV provider, or alternatively, to meet different medical, psychiatric, and social needs particularly during periods of extreme life instability (e.g. in the setting of homelessness or active drug use). [5]–[7]

Individuals transitioning from the criminal justice system to communities are a vulnerable segment of society with a disproportionately high prevalence of HIV, [8] substance use disorders [9], [10] with associated overdose and death, [11], [12] mental illness, [13], [14] and victimization. [15] Despite excellent virological control of HIV achieved while incarcerated, many of these benefits are lost upon release for reasons that have not yet been fully elucidated.[16]–[18] Multiple factors contribute to poor post-release outcomes for HIV-infected prisoners transitioning to the community. [19] One plausible explanation for loss of virological control after release is problems accessing routine healthcare. [20] Among Texas prisoners, only 5% of PLWHA prescribed antiretroviral therapy (ART) filled their prescriptions within 10 days post-release [21] and only 28% enrolled in an HIV clinic within one month. [22] Without access to and engagement in comprehensive care, released prisoners experience high rates of relapse to substance use, [16], [17] recidivism to prison or jail, [23] and reduced adherence to ART with loss of viral suppression. [16], [17] As the ED is often the safety net provider for those without regular access to primary care services, understanding ED use in this vulnerable population is important for optimizing HIV treatment and other health-related outcomes.

The objective of this study was to better understand factors associated with ED use among PLWHA in the year following release from prison. We calculated the 12-month event rate and correlates of ED use among HIV-infected released prisoners on ART. Our primary outcomes of interest were based on the Behavioral Health Model for Vulnerable Populations [24], [25] of healthcare utilization and included quantity and type of ED visits. Because we expected that greater social and medical instability immediately following prison release would result in greater reliance on ED care, we additionally examined the time to first ED visit from baseline.

## Methods

This is an observational longitudinal study of ED use among a cohort of released prisoners with HIV, prescribed ART and enrolled in a randomized clinical trial of directly administered antiretroviral therapy (DAART) versus self-administered antiretroviral therapy (SAT). All participants provided written informed consent to have their healthcare records reviewed at all designated sites. Ethical approval for all procedures was provided by the Yale University School of Medicine’s institutional review board and the Connecticut Department of Correction Research Advisory Committee.

**Figure 1:**
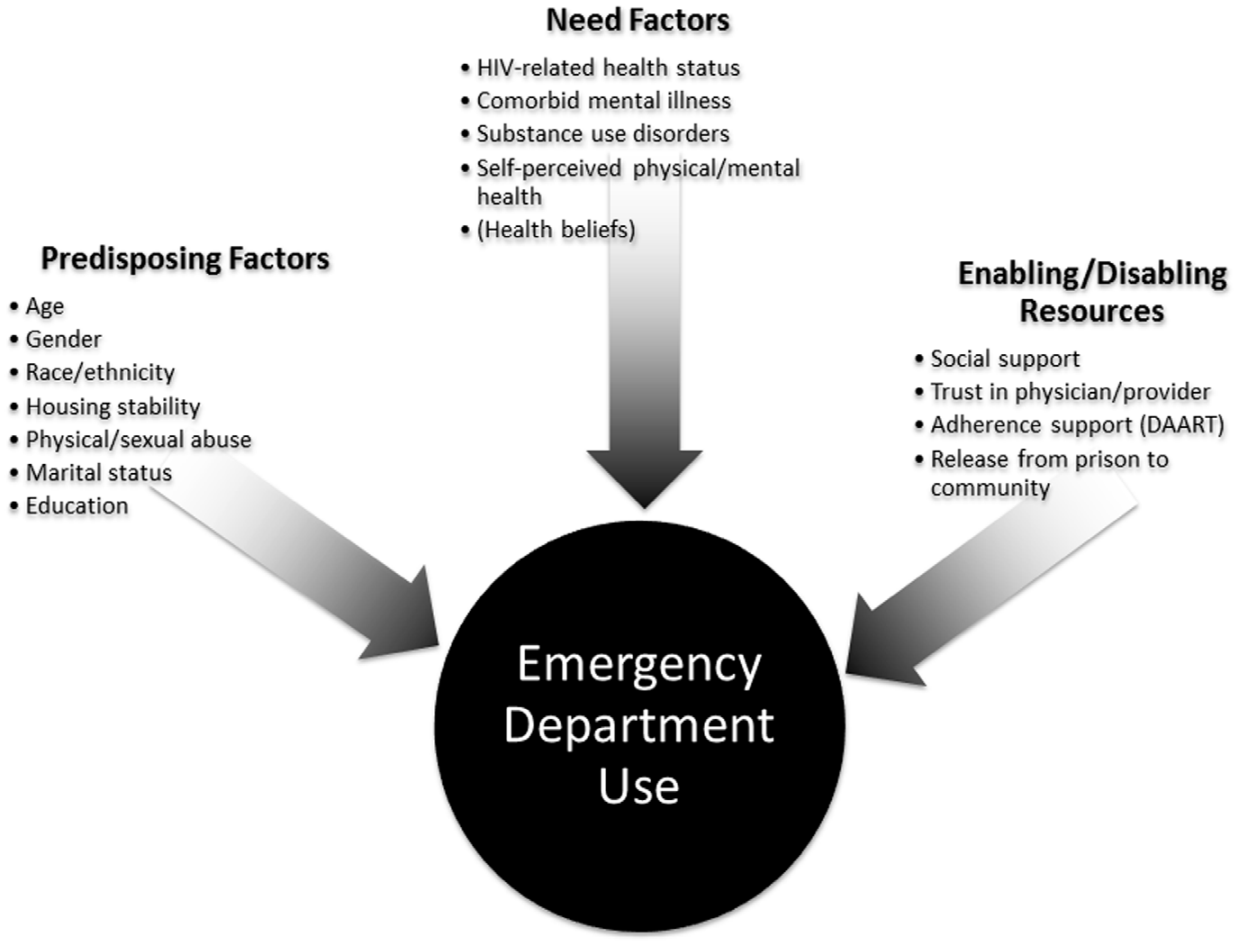
Conceptual Model of Determinants of Emergency Department Utilization. DAART  =  directly administered antiretroviral therapy.

The study’s analytical plan was based on the Behavioral Health Model [26], [27] that has been adapted for Vulnerable Populations. [24] This theoretical construct, depicted in Figure 1, proposes that healthcare utilization, including ED use, are driven by individual predisposing factors, enabling community resources, and need characteristics of populations and systems. Predisposing domains include demographic and social structures; enabling or disabling domains encompass personal, family or community resources; and need domains include perceived and objective measures of health, especially severity of disease. This model of healthcare utilization has been demonstrated to be applicable to homeless populations, [24] including HIV-infected persons released from jails, [28] who are socio-culturally marginalized in similar ways to released prisoners.

### Setting and Selection of Participants

The 151 study participants were released prisoners with HIV who were enrolled in a randomized clinical trial of DAART versus SAT between 2004 and 2009; details of the study and procedures are described elsewhere and registered at clinicaltrials.gov (NCT00786396). [29], [30] Eligibility criteria included age ≥18 years, laboratory-confirmed HIV infection, current receipt of ART, and planned return to Hartford or New Haven, Connecticut. Subjects were recruited from Connecticut prisons within 90 days of prison-release; however, community-referrals were allowed if the subject otherwise met eligibility criteria but were within 30 days after release to the community. In Connecticut, standard-of-care release procedures for prisoners with HIV involve receipt of a 14-day supply of all medications and standardized referral and appointments for continuity with patients’ community-based primary care providers. Of the 279 potential participants screened, 202 were considered eligible, and 154 were ultimately enrolled. [30] Reasons for exclusion were ineligibility (75 were not on ART, had a sentence modification, were no longer interested, or did not return to New Haven or Hartford) or non-enrollment (48 changed city, had a sentence modification, or were no longer interested in participating). [30] After informed consent, study participants were randomized 2∶1 to DAART or SAT and followed for 12 months after release; subjects were interviewed and assessed for HIV-1 RNA levels and CD4 counts quarterly. As part of the study, all participants received their ART from the AIDS Drug Assistance Program. Transitional case management additionally provided assistance with Medicaid enrollment and obtaining appointments at local HIV clinics. Adherence support was provided by trained outreach workers for subjects randomized to DAART and buprenorphine was offered if participants met DSM-IV criteria for opioid dependence and requested treatment. [31] Participants continued to receive all other healthcare from their primary providers. In previous randomized controlled trials of HIV-infected drug users, DAART was found to be superior to SAT in terms of HIV biological outcomes. [32].

### Study Protocol

As part of the baseline interview, participants signed a release of medical information for all ED facilities and other sites where they ever received or planned to receive medical care. Medical chart review was then conducted at the end of the 12-month period for enrolled participants at both EDs in New Haven and all three in Hartford to ensure complete coverage of each urban center and fully document the spectrum of ED utilization. The Veteran’s Administration (VA) and psychiatric hospitals were not accessed for chart review (although all psychiatric units within EDs were included and are a conduit to psychiatric inpatient care); none of the subjects identified the VA hospital as their primary site of care. Charts were included from the date of prison release for the 12 months post-release. Medical records were reviewed for all enrolled participants, regardless of attrition in the parent study, with ED visits otherwise concurrent with clinical trial participation. Chart reviews were conducted in accordance with methods proposed by Gilbert et al. [33] Using standardized forms, a trained reviewer extracted the following information from ED records, most of which were electronic: time and status of triage and discharge, patient chief complaint on triage, provider diagnoses and associated diagnostic codes, results of urine toxicology tests, whether the ED visit resulted in hospitalization (including psychiatric hospitalization), disposition from the ED, and discharge medications. There were no illegible charts and as much data as possible was recorded for each visit; all visits were included in the final analysis. All chart reviews were performed and reasons for visit coded by one author (JPM), after which 15% of charts were randomly reviewed by a second author (GLL) and discrepancies were resolved by a third author (FLA.).

### Methods of Measurement

The primary outcomes of interest were quantity and type of ED visits. Reasons for visit were based on the subjects’ triage chief complaints (grouped by involved organ system) and provider diagnoses (all of which fit into the following major exclusive categories: mental illness, trauma, substance abuse, social/access problems, acute/febrile syndromes, or somatic syndromes that, for the purposes of this analysis, included any issues related to acute or chronic pain.).

Unless noted, all other measurements were collected as part of the baseline comprehensive assessment.

#### Demographics

Baseline demographic parameters were assessed as part of the previously validated [34] Addiction Severity Index (ASI), 5^th^ Edition [35] including age, self-reported race/ethnicity, gender, education, marital status, and annual income. Anticipated housing status was categorized based on the question: *“How would you describe your expected living situation on the day of release?”* Subjects were considered homeless if they answered *“in a shelter”* or *“street or other public place;”* temporarily housed if they reported *“with a family member or friend temporarily,“ “haven’t decided yet,” “short term boarding,”* or *“drug treatment program;”* and permanently housed if they answered *“with a family member or friend permanently,”* or *“my own place.”*

#### Surrogates of HIV biological severity

Absolute CD4 count and quantitative HIV-1 RNA levels were measured at baseline and at 3-month follow-up. These two time points were chosen because data was available for the majority of participants and results reflected the initial destabilizing post-release period. CD4 count (cells/µL) was described as continuous and dichotomous (<200 vs. ≥200) variables; viral suppression was dichotomously defined as having an HIV RNA viral load <400 copies/ml.

History of abuse was measured on the ASI by self-report of any emotional, physical or sexual abuse in one’s lifetime. These questions have been shown to be a highly sensitive screen for subjects with histories of substance use and post-traumatic stress disorders (sensitivity  = 0.91) [36] Abuse was coded as a dichotomous variable indicating lifetime history of any physical or sexual abuse vs. none.

#### Mental illness

Depressive symptoms were measured using the 16-item Quick Inventory of Depressive Symptomatology Self-Report (QIDS-SR) (α = 0.86, 0.73–0.92). [37] Scores were described continuously from 0 to 27 to measure depression severity and also classified dichotomously as major depression (score >11) vs. no or mild depression (score ≤11).

#### Substance use disorders

Alcohol use disorders were measured using the Alcohol Use Disorders Identification Test (AUDIT) (α = 0.83, 0.75–0.97) with hazardous drinking defined as a score of ≥8 for men and ≥4 for women. [38], [39] Lifetime alcohol and drug use was measured by the ASI and severity was assessed using previously validated continuous alcohol and drug composite sub-scores that range from 0–1 with larger values reflecting greater severity. [35] If the subject initiated buprenorphine, this was recorded as a dichotomous variable.

#### Other health-related factors

Trust in physician was measured continuously using the 11-item Trust in Physician scale (α = 0.90). [40] Each item is scored on a 5-point Likert scale and a summary measure is equal to the unweighted mean of the responses (range 0–100 with higher scores reflecting higher levels of trust.) [41] Social support was measured continuously using the validated 31-item Social Support Scale (range 0–100 with higher scores reflecting greater social support). [42] HIV-associated quality of life was measured using the 36-item Medical Outcomes Study Short Form (SF-36) (α>0.80); [43] physical and mental health-related quality of life sub-scores were calculated separately using norm-based scoring (mean  = 50, standard deviation  = 10) with higher scores representing perception of better health.

### Statistical Analysis

Descriptive statistics were applied to the total cohort, stratified by number of ED visits. Using Kaplan-Meier survival curve estimates with Cox proportional hazard ratios, we looked at time to first ED visit focusing on different social and medical instability factors (gender, study arm, lifetime history of abuse, and housing stability). Statistical significance was determined by log rank tests; individuals without any ED visits were censored. Because of documented associations between histories of physical/sexual abuse with frequent ED visits for somatic complaints or episodic diseases, [44]–[46] a univariate analysis explored associations between lifetime histories of abuse and each of the categories of patients’ reasons for visit. To address non-Gaussian distribution of ED count data, Poisson regression was applied in multivariate models to determine the correlates of having any ED visit. Exposure time, defined as up to 12 months from study enrollment, was included as an offset variable. Multiple variables were evaluated for interaction or colinearity and none were found to be statistically significant. Based on the conceptual framework illustrated in Figure 1, we performed separate multivariate analyses of predisposing factors, need factors and enabling/disabling resources, including age, gender, and ethnicity in each model *a priori.* A multiple Poisson regression model was then determined by a forward stepwise elimination procedure, including all covariates illustrated in Figure 1 with *p*<0.05 Akaike’s Information Criterion (AIC) and Bayesian Information Criterion (BIC) were used to compare the multivariate models considered. [47], [48] Incidence rate ratios with 95% confidence intervals are reported and *p*-values <0.05 are considered to indicate a significant difference. To evaluate ED visits likely related to lower medical or psychiatric acuity, a multivariate analysis examined any ED use that did not result in hospital admission using Poisson regression and including variables with *p*<0.05 by forward stepwise selection. All analyses were performed with statistical software STATA version 9.0 (StataCorp, College Station, TX).

## Results

### Participant Characteristics

Of the 154 subjects enrolled in the parent trial, 130 were enrolled within 90 days pre-release and 24 were referred from the community within 30 days after release. Three subjects were excluded from the present analysis because they did not complete a full baseline interview or did not allow release of medical information, resulting in 151 evaluable subjects. Table 1 describes baseline characteristics of the total cohort and subjects with varying numbers of ED visits.

**Table 1:** Baseline Characteristics of Subjects Stratified by Number of Emergency Department Visits, n (%) or mean (SD).

	No ED visits (n = 66)	1 ED visit (n = 40)	>1 ED visits (n = 45)	>2 ED visits (n = 22)	Total Sample (n = 151)
Total number ED visits	0	40	187	141	227
Mean number ED visits per person-year	0	1.0 (0)	4.2 (3.2)	6.4 (3.4)	1.5 (2.5)
***Predisposing Factors***					
Mean age, years	45.2 (7.7)	46.6 (6.3)	45.3 (6.3)	46.8 (5.5)	45.6 (6.9)
Gender					
Male	59 (89.4%)	32 (80.0%)	32 (71.1%)	17 (77.3%)	123 (81.5%)
Female	7 (10.6%)	8 (20.0%)	13 (28.9%)	5 (22.7%)	28 (18.5%)
Mean years of education	10.6 (2.1)	11.0 (2.0)	10.7 (2.2)	10.9 (1.7)	10.8 (2.1)
Race or Ethnicity					
White	7 (10.6%)	4 (10.0%)	9 (20.0%)	6 (27.3%)	20 (13.3%)
Black	34 (51.5%)	24 (60.0%)	23 (51.1%)	11 (50.0%)	81 (53.6%)
Hispanic	25 (37.9%)	12 (30.0%)	13 (28.9%)	5 (22.7%)	50 (33.1%)
Marital status					
Married	5 (7.8%)	3 (7.7%)	5 (11.9%)	2 (10.0%)	13 (9.0%)
Never married	21 (32.8%)	12 (30.8%)	12 (28.6%)	4 (20.0%)	45 (31.0%)
Widowed/divorced	38 (59.4%)	24 (61.5%)	25 (59.5%)	14 (70.0%)	87 (60.0%)
Anticipated housing status					
Homeless	14 (22.6%)	7 (18.0%)	17 (37.8%)	12 (54.6%)	38 (26.0%)
Temporarily housed	36 (58.1%)	24 (61.5%)	19 (42.2%)	7 (31.8%)	79 (54.1%)
Permanently housed	12 (19.3%)	8 (20.5%)	9 (20.0%)	3 (13.6%)	29 (19.9%)
Lifetime history of physical or sexual abuse	25 (37.9%)	20 (50.0%)	24 (53.3%)	10 (45.5%)	69 (45.7%)
Study site					
Hartford	35 (53.0%)	21 (52.5%)	22 (48.9%)	10 (45.5%)	76 (50.3%)
New Haven	31 (47.0%)	19 (47.5%)	23 (51.1%)	12 (55.5%)	75 (49.7%)
***Need Factors***					
Mean baseline CD4 count	419.7 (247.7)	367.7 (228.2)	365.3 (234.2)	323.5 (194.0)	389.7 (238.6)
Baseline CD4 count <200 cells/uL	11 (16.7%)	12 (30.0%)	12 (26.7%)	6 (27.3%)	35 (23.2%)
Baseline HIV-1 RNA <400 copies/mL	56 (84.9%)	31 (81.6%)	31 (70.5%)	15 (71.4%)	118 (79.7%)
3-month HIV-1 RNA <400 copies/mL	45 (68.2%)	25 (62.5%)	22 (48.9%)	11 (50.0%)	92 (60.9%)
Hazardous Drinking	25 (38.5%)	14 (35.9%)	19 (42.2%)	11 (50.0%)	58 (38.9%)
Mean Lifetime Drug ASI score	0.167 (0.195)	0.188 (0.186)	0.161 (0.215)	0.235 (0.260)	0.171 (0.198)
Mean Lifetime Alcohol ASI score	0.223 (0.121)	0.236 (0.152)	0.254 (0.147)	0.219 (0.130)	0.235 (0.137)
Major Depression	21 (32.8%)	11 (29.7%)	19 (42.2%)	12 (54.6%)	51 (34.9%)
Mean SF-36 Physical Health composite score	49.2 (11.1)	46.3 (11.5)	40.9 (12.7)	39.5 (12.3)	45.9 (12.2)
Mean SF-36 Mental Health composite score	46.1 (14.0)	46.1 (13.3)	43.9 (14.4)	42.4 (15.0)	45.4 (13.9)
***Enabling/Disabling Resources***					
Mean Social Support score	64.2 (24.5)	62.9 (21.4)	64.8 (23.4)	62.7 (22.6)	64.0 (23.2)
Mean Trust in Physician score	67.6 (6.2)	67.8 (5.9)	67.8 (6.4)	68.4 (7.0)	67.8 (6.1)
Randomized to DAART	45 (68.2%)	23 (57.5%)	32 (71.1%)	17 (77.3%)	100 (66.2%)
Prescribed Buprenorphine	25 (37.9%)	9 (23.7%)	16 (35.6%)	8 (36.4%)	50 (33.6%)

ED =  emergency department; DAART  =  directly administered antiretroviral therapy; SD =  standard deviation; ASI  =  Addiction Severity Index; SF-36 =  Medical Outcomes Study Short Form.

Reflective of the statewide population of prisoners, [49] subjects were predominantly men (82%) and self-identified as black or Hispanic (87%) with 11±2 mean years of education and negligible preceding-year annual legal income (data not shown). Nearly 80% faced homelessness or being temporarily housed upon release. This included 37 participants (25% of the total cohort) who planned to stay in a shelter, one (0.7%) on the street or other public place, 51 (35%) with a family member or friend temporarily, 16 (11%) who were undecided, three (2%) in short term boarding, and nine (6%) in a drug treatment program. At the time of release, the majority of subjects (80%) had achieved viral suppression.

### Emergency Department Utilization

During the 12-month post-release period, 85 subjects visited the ED a total of 227 times, as depicted in Table 1. Over half (56%) of the total cohort visited the ED at least once and 15% frequented the ED more than twice per person-year. Principal reasons for visits, differentiating patients’ reasons for visit from ED provider diagnoses, are described in Table 2. The majority of visits were for episodic/febrile syndromes, pain, substance misuse issues, trauma or accidents, and decompensated mental illness. None of the univariate associations between abuse and types of ED visits were found to be statistically significant (data not shown).

**Table 2:** Patients’ Reasons for Visit and Provider Diagnoses for 227 Emergency Department visits.

Principal Reasons for Visit	Includes	Subject ComplaintsNo. visits (%)	Provider DiagnosisNo. visits (%)
Respiratory problems	Shortness of breath, cough, hemoptysis	34 (15%)	–
Extremity symptoms	Upper or lower extremity pain or swelling	31 (13.7%)	–
Neurologic problems	Altered mental status, agitation, seizures, syncope, loss of consciousness, lethargy, “found down”	30 (23.6%)	
Abdominal problems	Abdominal pain or swelling	23 (10.1%)	
Dermatologic problems	Rashes, bites, abscesses	18 (7.9%)	–
Mental illness	Depression, suicidal/homicidal thoughts, hallucinations, other psychotic symptoms	21 (9.3%)	25 (11%)
Back pain	Low back pain, back strain, lumbago	24 (10.6%)	–
Trauma	Injuries, accidents, sprains, hypothermia, dehydration, wounds	24 (10.6%)	41 (18.1%)
Substance abuse issues	Acute intoxication, overdose, requesting detoxification	12 (5.3%)	44 (19.4%)
Social/access problems	Requesting medications or refills, “homeless,” “I need a placeto sleep”	11 (4.8%)	10 (4.4%)
Cumulative febrile syndromes	Acute infections, episodic diseases	–	74 (32.6%)
Cumulative somatic syndromes	Any acute or chronic pain	–	46 (20.3%)

Disposition from the ED included: 53 visits (23%) resulted in hospital admission, of which five involved direct transfers to an inpatient psychiatric unit. Seven subjects were released from the ED back into police custody. Twelve visits were considered incomplete either because the patient left the ED against medical advice or left before being seen by an ED provider.

**Figure 2:**
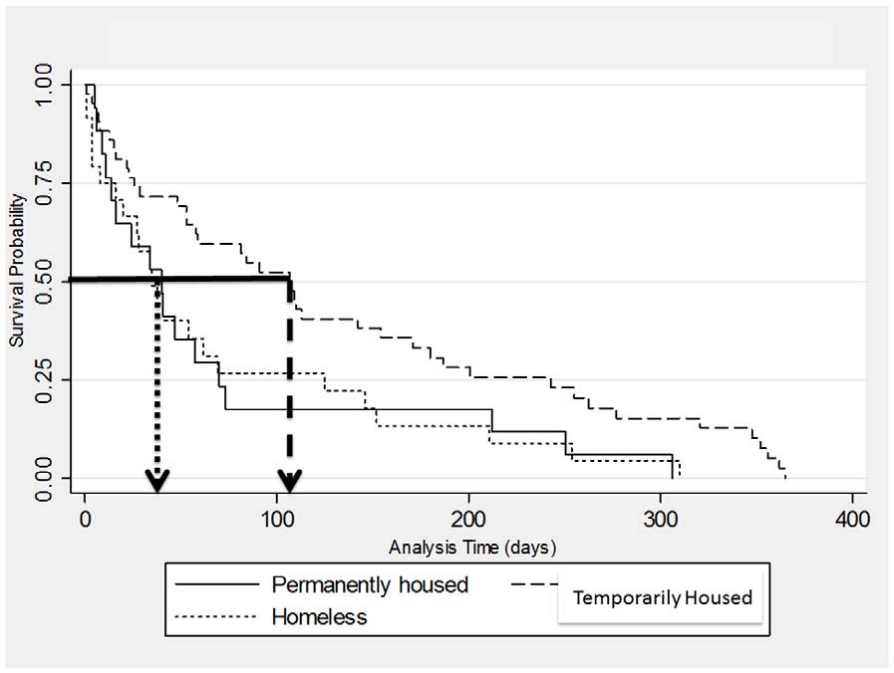
Kaplan-Meier Estimates of Time to First Emergency Department Visit by Anticipated Housing Status. Median for permanently housed (n = 29): 40 days Median for temporarily housed (n = 79): 107 days Median for homeless (n = 38): 35 days *p* = 0.014

Figure 2 depicts the time from prison release to first ED visit, stratified and statistically different based on anticipated housing status. Kaplan-Meier analysis demonstrates that those who were permanently housed or homeless first visited an ED sooner (median 40 and 35 days, respectively) than did participants who were temporarily housed (mean 107 days, *p* = 0.014). There were no significant differences in time to first ED visit based on gender, randomization to DAART versus SAT, or history of lifetime abuse (data not shown).

### Correlates of Emergency Department Use

Table 3 shows multivariate correlates of any ED use in the year following prison release. The stepwise elimination model, which used various factors from the predisposing, need, and enabling/disabling domains, was found to be the best fit using AIC and BIC criteria. Participants who used the ED were more likely than non-ED users to be older, white, not married, and experiencing moderate or severe depression. Participants who used the ED expressed lower levels of trust in their physician, had poorer self-perceived physical health, and lower mean lifetime alcohol severity scores. Participants who used the ED were not significantly different from non-ED users in terms of any HIV biological outcomes. Those anticipating being temporarily housed upon release were nearly half as likely as housed subjects to use the ED.

**Table 3:** Multivariate Correlates of Any Emergency Department Use (n = 151), IRR (95% CI), *p*-value.

	Predisposing Factors	Need Factors	Enabling/Disabling Resources	Stepwise Elimination
***Predisposing Factors***				
**Age** *	1.04 (1.01–1.06), *p* = 0.003	1.04 (1.01–1.06), *p* = 0.002	1.04 (1.02–1.06), *p* = 0.001	1.04 (1.01–1.06), *p* = 0.003
**Gender** (female = ref)				
Male	0.93 (0.65–1.33), *p* = 0.70	0.85 (0.58–1.25), *p* = 0.40	0.92 (0.65–1.32), *p* = 0.66	
**Race/Ethnicity** (white = ref)				
Black	0.49 (0.35–0.70), *p*<0.001	0.46 (0.31–0.67), *p*<0.001	0.41 (0.30–0.58), *p*<0.001	0.43 (0.30–0.63), *p*<0.001
Hispanic	0.43 (0.28–0.65), *p*<0.001	0.39 (0.26–0.58), *p*<0.001	0.36 (0.25–0.52), *p*<0.001	0.35 (0.23–0.54), *p*<0.001
**Mean years of education** *	1.02 (0.65–1.10), *p* = 0.55			
**Marital status** (married = ref)				
Widowed/divorced	0.95 (0.57–1.59), *p* = 0.86	–	–	–
Never married	1.24 (0.77–2.01), *p* = 0.38	–	–	1.56 (1.16–2.11), *p* = 0.004
**Housing status** (housed = ref)				
Temporarily housed	0.69 (0.47–1.02), *p* = 0.07	–	–	0.54 (0.40–0.73), *p*<0.001
Homeless	1.24 (0.83–1.87), *p* = 0.29	–	–	–
**Lifetime Abuse**	1.05 (0.80–1.39), *p* = 0.72	–	–	–
***Need Factors***				
**3 month VL <400**	–	0.80 (0.60–1.07), *p* = 0.13	–	–
**Baseline VL <400**	–	1.63 (1.12–2.38), *p* = 0.01	–	1.43 (0.99–2.05), *p* = 0.05
**Baseline CD4<200**	–	1.14 (0.79–1.63), *p* = 0.49	–	–
**Depression**				
Moderate/severe	–	1.56 (1.13–2.15), *p* = 0.007	–	1.80 (1.35–2.40), *p*<0.001
**Mean SF-36 Physical Health Composite score** *	–	0.97 (0.96–0.98), *p*<0.001	–	0.97 (0.96–0.98), *p*<0.001
**Mean SF-36 Mental Health Composite score** *	–	0.99 (0.98–1.00), *p* = 0.10	–	–
**Mean Lifetime Alcohol ASI score** *	–	0.42 (0.15–1.13), *p* = 0.09	–	0.21 (0.07–0.60), *p* = 0.004
**Mean Lifetime Drug ASI score** *	–	1.47 (0.70–3.10), *p* = 0.32	–	–
***Enabling/Disabling Resources***				
**Study Arm**				
DAART	–	–	1.07 (0.80–1.43), *p* = 0.66	–
**Mean Trust in Physician score** *	–	–	0.99 (0.97–1.01), *p* = 0.38	0.95 (0.93–0.98), *p*<0.001
**Mean Social Support score** *	–	–	1.00 (0.99–1.01), *p* = 0.70	1.01 (1.00–1.01), *p* = 0.02
**AIC**	601.1911	571.5224	634.4789	521.3184
**BIC**	633.6275	610.0394	658.4566	556.1817

DAART  =  directly administered antiretroviral therapy; VL =  viral load; ASI  =  Addiction Severity Index;

* For binomial variables, 0 is the referent group.

Multivariate correlates of the 174 ED visits that did not result in hospital admission are shown in Table 4. Subjects who were white, homeless, and had viral suppression at baseline were all more likely to have had ED visits that did not result in hospital admission. Those with higher lifetime alcohol addiction severity were less likely to have an ED visit not resulting in hospitalization. These subjects also reported poorer self-perceived physical and mental health with less trust in their physician.

**Table 4:** Multivariate Correlates of 174 Emergency Department Visits Not Resulting in Hospital Admission (n = 151).

	IRR (95% CI), *p*-value
**Housing status** (housed = ref)	
Homeless	1.55 (1.10–2.17), *p* = 0.01
**Mean Lifetime Alcohol ASI Score**	0.08 (0.02–0.30), *p*<0.001
**Race/Ethnicity (white = ref)**	
Black	0.44 (0.29–0.65), *p*<0.001
Hispanic	0.29 (0.18–0.46), *p*<0.001
**Viral Suppression at Baseline (VL <400)**	1.71 (1.11–2.63), *p* = 0.01
**Mean Trust in Physician score** *	0.95 (0.92–0.98), *p* = 0.002
**Mean SF-36 Physical Health Composite score** *	0.97 (0.96–0.98), *p*<0.001
**Mean SF-36 Mental Health Composite score** *	0.98 (0.97–0.99), *p*<0.001

ASI  =  Addiction Severity Index;

* For binomial variables, 0 is the referent group.

## Discussion

To our knowledge, this represents the first analysis of ED use among released prisoners with HIV on ART. The period of transition from prison to communities has been shown to be destabilizing, [11], [23], [50], [51] and our empiric study of ED use provides additional evidence of medical, psychiatric, and social vulnerability during this transition period. This study substantially adds to existing published literature by illustrating how ED use is often driven by psychiatric and social instability after prison release, even for individuals with well-controlled HIV.

Although our sample was relatively small, our findings are likely applicable to other populations of released prisoners with HIV in the U.S. A recent retrospective study in Rhode Island, for example, found significantly higher rates of psychiatric and substance use-related ED visits by released prisoners overall as compared to a general adult population. [52] Adding to this existing literature about the post-incarceration period, [53] our results further demonstrate the powerful impact of substance use co-morbidities, specifically drug or alcohol relapse, on ED use during this transition period. High prevalence of active substance use is reflected in our finding that 19% of ED visits were primarily for intoxication. Moreover, a significant proportion of visits were for trauma, wounds, accidents, exposure, and homelessness that are often indirectly related to active substance use.[54]–[56] Surprisingly, greater lifetime severity of alcohol use disorders was protective against ED visits, perhaps because individuals with long-term alcohol misuse are either socially isolated or are “functional” in spite of their substance use. Our data suggest that active intoxication and drug/alcohol relapse are more important markers of healthcare utilization than lifetime substance use severity. Hazardous drinking was not found to be associated with ED use in univariate or multivariate models. Post-incarceration, individuals may not have had time to relapse to alcohol use at a level of severity equal to that of baseline. While hazardous drinking was not significantly associated with increased ED use, more severe alcohol use disorders (e.g. harmful drinking, alcohol dependence) may be, and is a topic that warrants further exploration in future studies.

Overall, in our cohort of released prisoners with HIV, social instability factors are disproportionately represented reasons for visits compared to the general U.S. adult population in the most recent national surveys on ED use. [57] It was somewhat unexpected, therefore, that temporarily housed participants had a significantly longer time to first ED visit than their housed or homeless counterparts and that they were half as likely as housed subjects to visit the ED. In other words, extremes of housing stability (i.e. being either homeless or permanently housed) were associated with ED visits sooner after release from prison. This counterintuitive finding may be related to the fact that the majority of temporarily housed were at least staying with family or friends and therefore experienced less social isolation, which appears to have had a protective effect against ED visits immediately following prison-release. It might appear to be the case that the temporarily housed group, based on our definition, included a disproportionate number of individuals entering drug treatment, but sub-analyses confirm that those entering treatment included only nine subjects and being temporarily housed remained protective even after removing them from the analysis.

Regardless of housing stability, HIV-infected released prisoners overall are not dissimilar to other community-based vulnerable populations with HIV in terms of their heavy reliance on EDs for care. One recent study confirmed that “hard-to-reach” HIV populations, including the medically indigent, commercial sex workers, active substance users, homeless persons and prisoners, were significantly more likely to have had two or more ED visits in the prior 6 months (19.3% vs. 12.3%) when compared to a random sample of PLWHA. In another study, injection drug users in community settings having access to and utilizing low-threshold health services on a mobile health van reduced ED use, especially for those who were HIV-infected and had underlying psychiatric co-morbidity. [58] Importantly, these “hard-to-reach” individuals experienced excessive housing instability, unemployment, prior heroin/cocaine use, and mental illness. [59] In other community-based samples, recent ED use has also consistently been associated with homelessness and untreated substance use disorders, social and medical co-morbidities faced disproportionately by released prisoners. [5], [6], [60] As a co-morbid condition, HIV itself may be a less important driver of healthcare utilization: participants in our study with HIV viral suppression at baseline were *more* likely to have an ED visit following release. This finding did not reach statistical significance in a step-wise multivariate model of ED use, though the study may have been underpowered to detect a difference.

Our finding that increased ED use is associated with social access issues, mental illness and active substance use highlights the importance of addressing these factors as released prisoners adjust to community life, often in a destabilizing environment, after imprisonment. Notably for future research, these issues are amenable to interventions that might include provision of permanent housing, treatment for substance use disorders, or linkages to psychiatric care after release. [61] Multiple possible explanations and interventions have been proposed to improve HIV treatment outcomes during the immediate post-release period. [19] Previous research has confirmed that effective treatment of opioid dependence using buprenorphine during the 3-months post-release resulted in stabilized HIV treatment outcomes. [31] Another recent study further demonstrated that retention in buprenorphine treatment was significantly associated with reduced ED use in a cohort of opioid dependent patients accessing mobile healthcare services. [62] Though findings here did not demonstrate an impact of buprenorphine prescription on ED use, this may be explained in part because ED utilization appears to be more of a function of retention on buprenorphine, rather than receipt of a prescription. [62] In this sample, recent data confirm that retention on buprenorphine at 12 months was only 48%, but was associated with viral suppression. [63] Contributors to this low retention rate should be explored in future studies. The extent to which effective treatment of other substance use disorders affects HIV outcomes, however, has yet to be studied. [10] Ideally, such interventions would shift healthcare utilization from repetitive, episodic care provided in an ED and towards continuous primary and preventative care or, alternatively, to facilitate ED provision of a broader array of resources to those patients with highly comorbid social and psychiatric needs.

The finding that participants with higher lifetime alcohol use severity were less likely to use the ED overall seems counterintuitive, yet among this group that used the ED, their ED visit was significantly more likely to result in hospitalization. This complements national-level findings that alcohol-related ED visits are four times more likely to result in hospitalization compared to non-alcohol-related ED visits. [54] Overall, 23% of all ED visits in this study resulted in hospital admission, a rate almost double that of the general U.S. emergency patient population. [57] In fact, it is a rate comparable to that reported in studies of frequent ED users with 6–20 annual visits. [64] The decision to admit any patient from the ED to the hospital is certainly multi-factorial and may be based as much on availability of hospital- or community-based resources as on medical acuity. The significantly high rates of hospital admission in this cohort, however, attest to the great social, psychiatric, and medical needs of this patient population resulting in high demands placed on healthcare systems.

### Limitations

This study is a comprehensive assessment of ED use by released prisoners with HIV but represents the lower limits of ED use. All participants were on ART and two-thirds were receiving an intensive adherence intervention with DAART. Generalizability is limited by the relatively small population size and by the nature of participants’ involvement in the RCT which provided increased attention and oversight. Although participants were concurrently enrolled in a RCT that included transitional case management, case management has not been shown to significantly affect rates of ED or urgent care clinic utilization in other studies of released prisoners with HIV. [65] Participants may have used EDs outside of these two urban centers, and we may therefore have inadvertently missed visits by more geographically and socially transient patients; however, previous studies on ED use in Connecticut [58] suggest that our data are likely to account for the overwhelming majority of ED visits. Subjects re-incarcerated during the study period may have experienced relatively restricted ED utilization, reflective of either decreased active drug use or provision of onsite primary care in a correctional setting. [17], [23], [51] Data from Texas, however, suggests that just 20% of released prisoners with HIV are re-incarcerated within three years. [23] Baseline assessments of subjects reflect a single time point and may not reflect variations in status over the 12 months of observation. These assessments are what is known as a person transitions to the community and may provide guidance *a priori* for interventions that might reduce ED use after prison release. In addition, we did not have access to information about participants’ concurrent use of primary care, which may have confounded ED use. Last, this study lacks a control group; application of these findings to HIV-uninfected released prisoners or to other geographic regions where drug use patterns of or available support services for released prisoners differ must be interpreted with caution.

In spite of these limitations, this study has several important strengths in its design. Subjects’ healthcare use was followed for up to one year after prison release regardless of attrition in the parent study. This allowed us to track individuals in the community for whom the only point of contact with the healthcare system was the ED. Second, much of the health services literature relies on self-reported ED use, which may be skewed by social desirability or recall biases. In a population of released prisoners, there is often a prevailing mistrust of the healthcare system that makes self-report of healthcare utilization even less reliable. In contrast, our study objectively and comprehensively measured ED utilization based on medical chart review at all major EDs in the two urban centers of interest. Future research on this topic should include a control group of HIV-infected released prisoners receiving no intervention to better distinguish the impact of case management and adherence support on healthcare utilization patterns. From a health services perspective, future studies might explore ED use by including PLWHA who do not interface with the criminal justice system or people recently released from prison but not infected with HIV. We would exercise caution about interpreting such a study, however, because of the specific healthcare needs of PLWHA, the public health implications for retaining people in HIV care and on ART, and the unique destabilizing period that occurs during transition from prisons to communities.

### Conclusions

In this study of healthcare utilization by PLWHA prescribed ART and recently released from prison, ED use was highly correlated with mental illness, active substance use and extremes of housing stability, but not with HIV-related medical illness. These co-morbid illness and social factors should be addressed by future interventions because, as illustrated by the ED visits described here, substantial need exists.
